# A Prospective Observational Study of Maternal Near-Miss Cases at a Tertiary Care Center

**DOI:** 10.7759/cureus.105515

**Published:** 2026-03-19

**Authors:** Aparna S Patil, Aruna M Biradar, Laxmi Sangolli, Neelamma Patil, SR Bidri, Preeti Malapure, Apoorva Tippabhotla, Sarvada Umerjikar, Syeda Atufiyat Amreen, Anuradha Khavekar

**Affiliations:** 1 Obstetrics and Gynaecology, Shri BM Patil Medical College, Hospital and Research Centre, BLDE (Bharatiya Lingayat Development Educational Association) (Deemed to be University), Vijayapura, IND

**Keywords:** intensive care, maternal near miss, obstetric complications, severe acute maternal morbidity, tertiary care hospital

## Abstract

Introduction: Maternal near-miss (MNM) reflects women who survive life-threatening obstetric complications and serves as a key indicator for the quality of maternal care. The present study aimed to analyze the clinical and sociodemographic characteristics, referral patterns, management practices, and maternal-fetal outcomes of severe acute maternal morbidity (SAMM) cases at a tertiary care center.

Materials and methods: This prospective observational study was conducted from January 2024 to June 2025 at a tertiary care hospital. Pregnant women and those within 42 days of termination/delivery of pregnancy who fulfilled World Health Organization (WHO) MNM criteria were included. Data on sociodemographic characteristics, obstetric profile, referral pattern, clinical features, management, and maternal and fetal outcomes were recorded.

Results: Among the 100 MNM cases, most women were aged 21-25 years (48 (48%)), resided in rural areas (65 (65%)), and were multigravida (69 (69%)). Anemia was highly prevalent, with moderate anemia in 45 (45%) and severe anemia in 36 (36%). Emergency lower segment cesarean section (LSCS) was the predominant mode of delivery (72 (73.4%)). Among the referred cases (n=77), district hospitals were the main source (49 (63.6%)), and 36 (46.7%) reached the tertiary center within 1 hour. Major obstetric complications included abruptio placentae (13 (13%)), HELLP (hemolysis, elevated liver enzymes, and low platelet count) syndrome (10 (10%)), and disseminated intravascular coagulation (DIC) (9 (9%)), while shock was observed in 20 (20%) cases. The most frequent WHO near-miss laboratory criterion was oxygen saturation <90% for >60 minutes (90 (90%)), and 48 (48%) women required transfusion of ≥5 units of red cell concentrate. Fetal outcomes (n=98) showed live births in 77 (78.5%) cases, low birth weight in 40 (40.8%), and neonatal intensive care unit (NICU) admission in 33 (37.9%).

Conclusion: MNM events in this study were commonly associated with severe obstetric complications, such as hemorrhage, hypertensive disorders, and anemia, and frequently required intensive medical interventions. Early recognition of high-risk pregnancies, timely referral, and strengthened critical care services may help reduce severe maternal morbidity and improve maternal and neonatal outcomes. However, the findings should be interpreted cautiously due to the descriptive design and single-center setting of the study.

## Introduction

A maternal near-miss (MNM), as defined by the World Health Organization (WHO), describes a woman who survived a life-threatening complication that occurred during pregnancy, childbirth, or within 42 days following termination of pregnancy, either because of chance or timely medical intervention [1,2]. Enhancement of maternal health remains a key priority within the Sustainable Development Goals (SDGs), which aim to reduce the global maternal mortality ratio (MMR) to below 70 per 100,000 live births by the year 2030 [3]. Despite substantial progress in maternal healthcare, maternal mortality remains unacceptably high worldwide, particularly in low-income countries where approximately 99% of maternal deaths are reported [4]. Because maternal mortality alone does not fully represent the overall burden of maternal health complications, MNM has gained recognition as an important indicator for assessing maternal health status and evaluating the quality of obstetric care [5].

Obstetric complications occurring during pregnancy and the intrapartum period continue to be major contributors to maternal morbidity and mortality in developing countries, including India [6]. Reported MNM ratios in India show considerable variation, ranging from 3.9 to 379.5 per 1,000 live births and 7.6 to 60.4 per 1,000 deliveries, with MNM-to-maternal mortality ratios reported between 1.7 and 21.8 [7]. Achieving the SDG target of reducing maternal mortality to less than 70 per 100,000 live births by 2030 requires focused attention on women who experience severe complications during pregnancy, childbirth, or the postpartum period [8,9]. Maternal deaths represent only the visible portion of the problem, whereas a much larger number of women survive severe life-threatening complications, forming the broader base of the “iceberg” of maternal morbidity [8,9].

Evaluation of maternal mortality and MNM events at the facility level can provide essential baseline data regarding the quality of obstetric care delivered [10]. The WHO has proposed standardized criteria for identifying MNM cases, which include clinical, laboratory, and management-based indicators reflecting organ dysfunction [11]. The “three delays model,” originally described by Thaddeus and Maine, offers an important framework for understanding the factors that contribute to adverse maternal outcomes: delay in deciding to seek care (Phase I), delay in reaching an appropriate healthcare facility (Phase II), and delay in receiving adequate treatment after reaching the facility (Phase III) [12]. The first two phases mainly represent demand-side barriers influenced by social, cultural, and economic factors, whereas the third phase reflects supply-side limitations such as inadequate resources, institutional inefficiencies, and delays in clinical management [12]. In many low-resource settings, women with severe obstetric complications often reach tertiary healthcare facilities in critical condition, and additional systemic delays in management may further worsen maternal outcomes [13].

Systematic monitoring and evaluation of MNM cases provide important insights into the effectiveness of referral systems, availability of life-saving interventions, and the overall quality of obstetric care services [14]. Unlike maternal deaths, near-miss cases enable direct assessment of the healthcare process and allow survivors to share their experiences, thereby offering valuable information for strengthening health systems. The concept of severe acute maternal morbidity (SAMM) and MNM events was introduced to complement maternal death reviews, particularly to address the limitations associated with the relatively lower frequency of maternal deaths [15]. An ideal MNM identification system should be easy to apply, capable of identifying women at the highest risk of death, and suitable for comparison across different healthcare settings. Evaluation of such cases contributes significantly to clinical audit, quality improvement initiatives, and evidence-based policy formulation.

Therefore, the present study aimed to analyze the clinical and sociodemographic characteristics, referral patterns, management interventions, and maternal-fetal outcomes among SAMM or MNM cases at a tertiary care hospital.

This research work was originally conducted as part of a postgraduate dissertation submitted to the Department of Obstetrics and Gynecology, BLDE (Deemed to be University), Vijayapura, Karnataka, India.

## Materials and methods

This prospective observational study was conducted from January 2024 to June 2025 in the Department of Obstetrics and Gynecology at a tertiary care hospital. Pregnant women within 42 days following termination/delivery of pregnancy, developing severe obstetric complications and requiring intensive management, were screened for inclusion after obtaining written informed consent. Women with morbidity due to non-obstetric causes were excluded. The study was approved by the Institutional Ethics Committee (IEC/875/2022-23).

The sample size was calculated using the formula $n=\left( Z^{2}\times p\times \left[ 1-p \right] \right)/d^{2}$, with Z = 1.96 for a 95% confidence interval, an assumed population proportion of 0.07 based on hospital data, and a margin of error of 0.05, yielding a required sample size of approximately 100. Accordingly, 100 MNM cases were included.

MNM cases were identified using the WHO near-miss criteria, which include severe maternal complications, critical interventions, and evidence of organ dysfunction [16]. Severe maternal complications included severe postpartum hemorrhage, severe preeclampsia, eclampsia, sepsis or severe systemic infection, ruptured uterus, severe complications of abortion, and ruptured ectopic pregnancy [16]. Critical interventions included admission to the intensive care unit (ICU), laparotomy other than cesarean section, and transfusion of five or more units of blood or blood products [16]. Organ dysfunction was defined using cardiovascular, respiratory, renal, hematological, hepatic, and neurological parameters as specified by the WHO criteria [16].

Data collection was carried out prospectively by the investigators through direct patient assessment, review of medical records, and laboratory reports during the hospital stay. All eligible patients were monitored from admission until discharge or death to document the complete clinical course and outcomes.

For all eligible cases, demographic and obstetric details such as age, residence, parity, booking status, and gestational age were recorded. Clinical data, including antenatal history, referral source, referral status (self-referred or referred from peripheral facilities), time taken to reach the hospital, and transport-related problems were documented. Findings from physical examination and relevant laboratory investigations were noted. Management details, including operative procedures, blood transfusion, ventilatory support, use of vasoactive drugs, dialysis, and ICU care, were recorded. Maternal complications, fetal outcomes, and duration of ICU and hospital stay were documented, and patients were followed until discharge.

Data were entered into Microsoft Excel (Microsoft, Redmond, WA) and analyzed using the Statistical Package for the Social Sciences (SPSS) version 26 (IBM Corp., Armonk, NY). Descriptive statistical analysis was performed. Categorical variables were expressed as frequencies and percentages (N (%)), and the analysis was limited to descriptive statistics as the study aimed primarily to describe the clinical profile, management patterns, and outcomes of MNM cases.

## Results

During the study period, 3242 women were admitted to the labor ward, among whom 100 MNM cases were identified. Among 100 MNM cases, the majority of women were aged 21-25 years (48 (48%)), followed by 26-30 years (37 (37%)). Most participants were housewives (92 (92%)) and resided in rural areas (65 (65%)). Multigravida constituted 69 (69%) cases, and 75 (75%) were booked antenatal cases (Table 1).

**Table 1:** Sociodemographic and Obstetric Characteristics (n = 100) Data are presented as N (%). MNM, maternal near-miss.

Variable	Category	N (%)
Age (years)	<20	8 (8%)
	21-25	48 (48%)
	26-30	37 (37%)
	>30	7 (7%)
Occupation	Housewife	92 (92%)
	Laborer	6 (6%)
	Tailor	2 (2%)
Residence	Rural	65 (65%)
	Urban	35 (35%)
Gravida	Primigravida	31 (31%)
	Multigravida	69 (69%)
Booking status	Booked	75 (75%)
	Unbooked	25 (25%)

Most deliveries occurred at BLDE Hospital (71 (71%)), and among delivered women (n=98), emergency lower segment cesarean section (LSCS) was the predominant mode (72 (73.4%)), followed by vaginal delivery (25 (25.5%)). Of the referred cases (n=77), the majority were referred from district hospitals (49 (63.6%)). Nearly half of the women reached the hospital within 1 hour (36 (46.7%)), and transport-related problems were reported in 7 (9.1%) cases (Table 2).

**Table 2:** Delivery and Referral Characteristics Data are presented as N (%). Mode-of-delivery analysis was performed among delivered women (n=98), while referral characteristics were assessed among referred cases (n=77). LSCS, lower segment cesarean section; PHC, primary health center; CHC, community health center.

Variable	Category	N (%)
Place of delivery	BLDE Hospital	71 (71%)
	District hospital	21 (21%)
	Private hospital/Home	8 (8%)
Mode of delivery (n=98)	Emergency LSCS	72 (73.4%)
	Vaginal delivery	25 (25.5%)
	Elective LSCS	1 (1.0%)
Referral source (n=77)	District hospital	49 (63.6%)
	Private/Nursing home	16 (20.7%)
	PHC/CHC	12 (15.5%)
Time to reach hospital (n=77)	≤30 minutes	20 (25.9%)
	1 hour	36 (46.7%)
	2 hours	18 (23.3%)
	≥3.5 hours	3 (3.8%)
Transport problem	Yes (ambulance unavailable)	7 (9.1%)

Fetal outcomes were assessed in 98 cases. Live births accounted for 77 (78.5%), while intrauterine deaths occurred in 21 (21.4%). Low birth weight (<2.5 kg) was noted in 40 (40.8%) neonates, and 33 (37.9%) required NICU admission, most commonly due to respiratory distress syndrome (28 (28%)). Male neonates comprised 52 (53.1%) births (Table 3).

**Table 3:** Fetal Outcomes (n=98) Data are presented as N (%). Fetal outcome analysis was performed only among women who delivered during the study period. NICU, neonatal intensive care unit.

Variable	Category	N (%)
Gender	Male	52 (53.1%)
	Female	46 (46.9%)
Outcome	Live birth	77 (78.5%)
	Intrauterine death	21 (21.4%)
Birth weight	<2.5 kg	40 (40.8%)
	≥2.5 kg	58 (59.2%)
NICU admission	Yes	33 (37.9%)
Indication for NICU	Respiratory distress syndrome	28 (28%)
	Preterm/Low birth weight	21 (21%)
	Asphyxia	1 (1%)

Anemia was the predominant maternal complication, with moderate anemia in 45 (45%) and severe anemia in 36 (36%) cases. Among obstetric complications, abruptio placentae was observed in 13 (13%), HELLP (hemolysis, elevated liver enzymes, and low platelet count) syndrome in 10 (10%), and disseminated intravascular coagulation (DIC) in 9 (9%) cases, representing the major contributors to maternal morbidity (Table 4).

**Table 4:** Maternal Complications Data are presented as N (%). HELLP, hemolysis, elevated liver enzymes, and low platelet count; DIC, disseminated intravascular coagulation; GDM, gestational diabetes mellitus; PROM, premature rupture of membranes.

Category	Complication	N (%)
Medical complications	Cardiac disorder	7 (7%)
	Epilepsy	4 (4%)
	Hypothyroidism	4 (4%)
	Bronchial asthma	1 (1%)
	Moderate anemia	45 (45%)
	Severe anemia	36 (36%)
	Acute renal failure	4 (4%)
Obstetric complications	Abruptio placenta	13 (13%)
	HELLP syndrome	10 (10%)
	Disseminated intravascular coagulation	9 (9%)
	Gestational diabetes mellitus	6 (6%)
	Gestational hypertension	5 (5%)
	Placenta previa	2 (2%)
	Premature rupture of membranes	1 (1%)

Under WHO near-miss criteria, respiratory dysfunction was prominent, with oxygen saturation <90% observed in 90 (90%) and PaO₂/FiO₂ <200 mmHg in 38 (38%) cases. Clinically, abnormal respiratory rate was noted in 28 (28%) and shock in 20 (20%) women. Management-based criteria revealed that 48 (48%) required transfusion of ≥5 units of red cell concentrate and 40 (40%) required intubation and ventilation, while 16 (16%) required continuous vasoactive support (Table 5).

**Table 5:** WHO Near-Miss Criteria (n = 100) Data are presented as N (%). The WHO near-miss criteria include clinical, laboratory, and management-based indicators of organ dysfunction. WHO, World Health Organization; PaO₂/FiO₂, ratio of arterial oxygen partial pressure to fractional inspired oxygen; RCC, red cell concentrate.

Category	Criterion	N (%)
Clinical criteria	Shock	20 (20%)
	Breathing rate >40/min or <6/min	28 (28%)
	Loss of consciousness	2 (2%)
	Gasping	10 (10%)
	Acute cyanosis	2 (2%)
	Coagulation disorders	2 (2%)
Laboratory criteria	Oxygen saturation <90% for >60 min	90 (90%)
	Creatinine >3.5 mg/dL	7 (7%)
	PaO₂/FiO₂ <200 mmHg	38 (38%)
	Platelets <50 × 10⁹/L	7 (7%)
	Bilirubin >100 µmol/L or >6.0 mg/dL	0 (0%)
	Lactate >5 mg/dL	0 (0%)
	pH <7.1	0 (0%)
Management criteria	Continuous use of vasoactive drugs	16 (16%)
	Transfusion ≥5 units red cell concentrate	48 (48%)
	Dialysis for acute kidney failure	5 (5%)
	Cardiopulmonary resuscitation	2 (2%)
	Intubation and ventilation >60 min	40 (40%)

The majority of women had an ICU stay of less than five days (90 (90%)). Hospital stay ranged between five and 10 days in 88 (88%) cases, while 12 (12%) required hospitalization beyond 10 days, indicating short-duration but intensive in-hospital management in most patients (Table 6).

**Table 6:** ICU and Hospital Stay Data are presented as N (%). ICU, intensive care unit.

Variable	Category	N (%)
ICU stay	<5 days	90 (90%)
	≥5 days	10 (10%)
Hospital stay	5-10 days	88 (88%)
	>10 days	12 (12%)

## Discussion

The present study demonstrates that MNM events were most common among women aged 21-25 years (48 (48%)), with a predominance of rural residents (65 (65%)) and multigravida women (69 (69%)). A substantial proportion were also anemic, with moderate anemia in 45 (45%) and severe anemia in 36 (36%), indicating the important contribution of maternal anemia and nutritional deficiencies to severe maternal morbidity. This high burden of anemia was reflected in the substantial requirement for blood and blood component therapy. Whole-blood transfusion was administered in 36% of women, while packed cell volume transfusion was required in the majority, with 27 (27%) receiving three units and 20 (20%) receiving two units. Fresh frozen plasma was administered in ≥4 units in 37 (37%) cases, and platelet transfusion was required in multiple units in a considerable proportion, most commonly two units in 19 (19%) women. The high transfusion requirement further underscores the severity of hematological compromise and the significant contribution of anemia and coagulopathy to MNM events. These findings reaffirm the vulnerability of young, multiparous women from rural backgrounds and highlight persistent gaps in antenatal optimization. Similar sociodemographic trends have been reported by Sayyed et al. [17] and Patankar et al. [18], who observed higher MNM rates among women from rural areas and those with delayed access to tertiary care facilities.

Although the majority of deliveries occurred in health institutions, emergency LSCS was the predominant mode of delivery (72 (73.4%)), reflecting the critical condition in which many women presented. Among the referred cases (n=77), district hospitals were the major source (49 (63.6%)). Among the referred cases, 36 (46.7%) reached within 1 hour. This suggests relatively prompt transport in most cases. However, the need for referral itself indicates limited capacity for managing severe obstetric complications at peripheral centers. Similar patterns of referral burden and delayed escalation of care have been documented in other low-resource settings [19-21].

In the present study, obstetric complications were more frequent than medical comorbidities. The leading obstetric complications included abruptio placenta (13 (13%)), HELLP syndrome (10 (10%)), DIC (9 (9%)), and gestational diabetes mellitus (6 (6%)), while gestational hypertension was noted in 5 (5%). Among medical disorders, cardiac disease (7 (7%)) was the most common, followed by epilepsy (4 (4%)), hypothyroidism (4 (4%)), and bronchial asthma (1 (1%)). Additionally, clinical manifestations of organ dysfunction, such as respiratory rate >40/<6 per minute (28 (28%)) and shock (20 (20%)), reflected the severity of presentation. These findings are consistent with the studies from Maharashtra and South Africa, where hypertensive disorders, coagulopathy, and hemorrhagic complications were leading contributors to MNM [22,23].

With respect to the WHO near-miss criteria, laboratory-based indicators predominated in our study. The most frequent criterion was oxygen saturation <90% for more than 60 minutes, observed in 90 (90%) cases, followed by PaO₂/FiO₂ <200 mmHg in 38 (38%). Clinical criteria such as shock (20 (20%)), abnormal respiratory rate (28 (28%)), and gasping (10 (10%)) were also notable. Among management-based criteria, transfusion of ≥5 units of red cell concentrate was required in 48 (48%), intubation and ventilation for >60 minutes in 40 (40%), and use of vasoactive drugs in 16 (16%) cases. These findings indicate that hypoxemia and the need for aggressive resuscitative measures were central features of MNM in our cohort. The predominance of respiratory dysfunction and high transfusion requirement underscores the multisystem involvement characteristic of severe maternal morbidity and aligns with the organ dysfunction framework proposed by WHO [16]. Similar distributions of laboratory and management criteria have been described in regional studies evaluating MNM [24].

Fetal outcomes further reflected the severity of maternal compromise. Among 98 assessed pregnancies, live births occurred in 77 (78.5%), while intrauterine deaths were seen in 21 (21.4%), low birth weight was observed in 40 (40.8%), and NICU admission was required in 33 (37.9%), most commonly due to respiratory distress syndrome. These findings emphasize the close interrelationship between severe maternal morbidity and adverse perinatal outcomes, consistent with previous reports by Chandran JR et al., demonstrating increased prematurity and neonatal intensive care utilization among MNM cases [25].

Strengths and limitations

The present study has several strengths. First, the prospective study design enabled real-time data collection and minimized recall bias, thereby improving the accuracy of clinical documentation and outcome assessment. Second, the use of the WHO MNM criteria provided standardized case identification, facilitating comparison with other studies. In addition, comprehensive data collection on sociodemographic characteristics, clinical features, referral patterns, interventions, and maternal and neonatal outcomes allowed a multidimensional assessment of MNM events. The conduct of the study in a tertiary care referral center enabled evaluation of severe obstetric cases and management practices, providing valuable insights into referral patterns and healthcare system challenges. Furthermore, the study contributes regional evidence from North Karnataka, particularly the Vijayapura region, where limited published data on MNM events are available.

However, several limitations should be acknowledged. The single-center design limits the generalizability of the findings to other healthcare settings. In addition, the relatively small sample size (n=100) may not capture less common causes of MNM events. The analysis was primarily descriptive, and no inferential statistical methods were applied to identify predictors or associations, which limits the ability to establish risk factors for MNM. Furthermore, the study did not include a detailed qualitative evaluation of health system delays or patient experiences, which could provide deeper insights into barriers to timely maternal care.

## Conclusions

This study highlights that MNM events remain an important indicator of severe maternal morbidity and continue to represent a significant public health concern in tertiary care settings. In the present study, MNM cases were commonly associated with obstetric complications such as hemorrhage, hypertensive disorders, and severe anemia, and a substantial proportion of women required intensive interventions, including blood and blood component transfusion, ICU support, and emergency surgical procedures, reflecting the severity of presentation. These findings emphasize the importance of early recognition of high-risk pregnancies, timely referral from peripheral healthcare facilities, and coordinated multidisciplinary management to prevent progression to maternal death. Strengthening antenatal screening, referral systems, and the availability of critical care services may contribute to improving maternal outcomes. However, as the study was descriptive and conducted at a single tertiary care center, the findings should be interpreted cautiously, and further multicenter analytical studies are warranted to better identify predictors of MNM events and guide targeted interventions.
